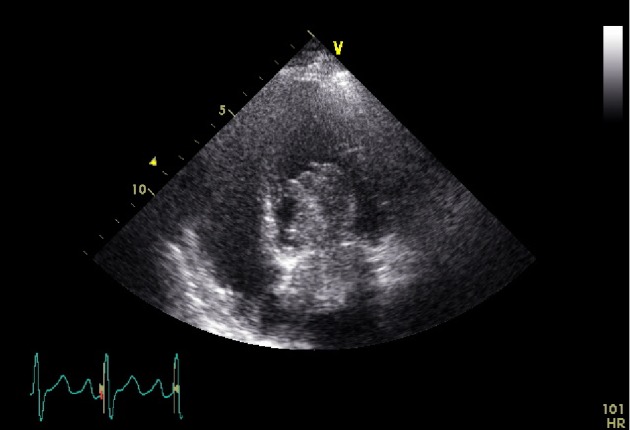# Anesthetic Management of a Voluminous Left Atrial Myxoma Resection in a 19 Weeks Pregnant with Atypical Clinical Presentation

**DOI:** 10.1155/2019/4181502

**Published:** 2019-12-12

**Authors:** Alexandros Alexis, Pierre Origer, Jean-Pierre Hacquebard, Didier De Cannière, Olivier Germay, Jean-Luc Vandenbossche, Yota Kapessidou

**Affiliations:** ^1^Department of Anesthesiology, University Hospital Saint-Pierre, Université Libre de Bruxelles (ULB), Brussels, Belgium; ^2^Department of Cardiac Surgery, University Hospital Saint-Pierre, Université Libre de Bruxelles (ULB), Brussels, Belgium; ^3^Department of Cardiology, University Hospital Saint-Pierre, Université Libre de Bruxelles (ULB), Brussels, Belgium

## Abstract

We report the case of a semi-urgent cardiac surgery, in a 19 gestation age pregnant. Despite the fact that the patient was asymptomatic, except for some palpitations, a large left auricle (LA) myxoma was fortuitously diagnosed with transthoracic echocardiography (TEE). Considering the important embolic risk, the tumor was successfully removed during cardiac surgery under cardiopulmonary bypass (CPB). Fetal bradycardia following defibrillation under stable maternal and CPB conditions was successfully managed. The postoperative period and remainder of the pregnancy was smooth and the delivery uneventful.

## 1. Introduction

Myxomas are rare benign tumors (0.5 per million population per year), that account for the 30–50% of all cardiac tumors and are most commonly located in the left atrium (>85%) [1]. Despite the female predominance, myxoma incidence during pregnancy is extremely low, with only 51 reported cases in the literature to date [2]. Patients have an increased risk of acute cardiogenic shock or sudden cardiac death given the potential for embolization and hemodynamic deterioration upon blood outflow obstruction [2]. Standard therapy involves complete surgical resection. However, cardiac surgery requiring cardiopulmonary bypass (CPB) during pregnancy is a high-risk, challenging procedure for anesthesiologists, associated with high maternal mortality (2.9–13.3%), mostly in emergency setting, and particularly high fetal mortality (14.3–38.5%) [3]. If surgery cannot be delayed until fetal maturation, fetal heart rate monitoring during CPB is recommended, when gestational age (GA) is greater than 24 weeks [4].

We report the case of a 19 weeks parturient undergoing cardiac surgery under CPB for the resection of a left atrial myxoma. Importance is given to the particularity of case's clinical presentation. The procedure was marked by extreme transient fetal bradycardia following defibrillation as revealed by continuous fetal heart rate monitoring. Special attention is given to the anesthetic management of such cases, due to their complexity, as reported in the literature.

## 2. Case Presentation

A 34-year-old woman, 19 weeks pregnant, G1P0, NYHA I, was referred to cardiologist for palpitations. She had minor medical and surgical history, one previous uneventful pregnancy and a second one with symptoms of preeclampsia. During the first trimester of pregnancy, both fetus and mother were in perfect health.

**Figure 1:**
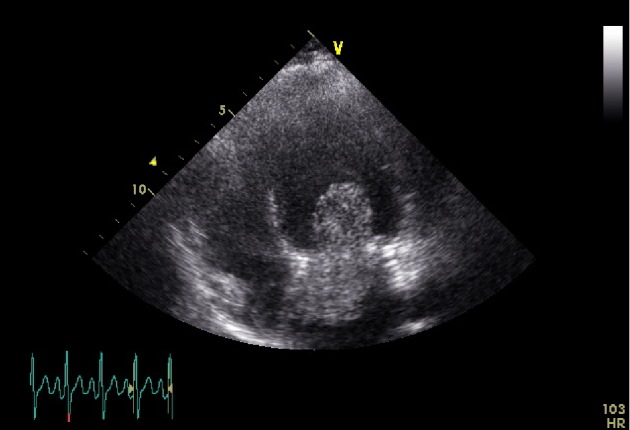


Transthoracic echocardiography (TTE) revealed a large (70 × 32 mm) plurilobulated, pedunculated tumor in the left atrium (Figures 1 and 2), attached to the interauricular septum. Mild mitral regurgitation was noted, due probably to the tumor protrusion into the mitral valve orifice at diastole. There were no evidence of left ventricular inflow obstruction or elevated pulmonary pressure and the ejection fraction was conserved. The image was suggestive of myxoma, and a multidisciplinary care coordination between obstetricians, cardiologists, cardiac surgeons, anesthesiologists, perfusionists and neonatologists took place in order to evaluate the surgical risk to perform cardiac surgery under CPB at this early GA (19 weeks). Considering the high embolic risk for the mother, a semi-urgent surgical resection using continuous intraoperative fetal heart rate monitoring of the mass was proposed to the patient. She did made an autonomous decision and consented to the procedure, despite the high risk of fetal loss.

Physical examination revealed an irregular rhythm without any additional murmurs and tame bilateral oedema of lower limbs. Heart rate was 108 beats per minute (bpm) and arterial pressure 130/80. Chest X-ray was normal and an electrocardiogram (ECG) indicated sinus rhythm with ventricular extrasystoles (96 bpm).

Upon arrival into the operating room, the patient was placed in a 15° left tilted supine position, a large-bore peripheral IV line was inserted and the standard monitoring for cardiac surgery of our institution was applied (5 leads ECG, femoral arterial catheter and state entropy).

General anesthesia of the patient, weighing 72 kg and measuring 162 cm, was induced after 3 min of preoxygenation and ventilation by means of a Sellick maneuver, using target controlled intravenous anesthesia (TCI) with a combination of Remifentanil (Minto model) and Propofol (Schneider model) to ensure a level between 40 and 60 state entropy. After intubation and mechanical ventilation set up, an ultrasound-guided right internal jugular catheter and a bladder catheter and rectal thermometer were placed. The PICCO II monitor was used to monitor cardiac output and other related hemodynamic data.

Mean arterial blood pressure (MAP) target prior to CPB was set at 70 mmHg. Concerning prevention strategy of bleeding during CPB, we did not use tranexamic acid, because of the lack of evidence about its effect on fetus. Anticoagulation therapy by heparin (400 U/kg) was administered before aorta cannulation.

Despite the technical difficulties related to the small fetal size, and its unpredictable position during surgery, mobile external continuous cardiotocography (EFM), under sterile conditions, was applied and surveyed by a midwife, throughout the whole procedure.

Surgery was initiated by a median sternotomy, and CBP was established using ascending aorta-right atrium (AA-RA) bicaval cannulation. Perfusion strategy consisted in pulsatile flow, ranging from 2.9 to 3.1 L·min^−1^·m^2^ (theoretic cardiac index = 2.8 L·min^−1^·m^2^) and high perfusion pressure (66–81 mmHg), under normothermic conditions (36-37°). A gradual transition from corporeal to fully extracorporeal circulation was realized. Fetal heart rate was not affected by the initiation of CPB. The perfusionist maintained hematocrite values greater that 28% in order to maximize fetal oxygen carrying. The heart was arrested by antegrade coronary perfusion with cold blood cardioplegia. Anesthesia level during CPB was adjusted according to state entropy (40–60).

The surgical procedure consisted in a vertical bi-atrial incision providing access to the smooth pedunculated mass, implanted in the fossa ovalis (FO). The mass was then excised and removed with its stalk en bloc. Then the atrial septal defect was closed using a pericardial patch and the left and right atrium were stitched.

After de-airing the cardiac cavities, the aortic clamp was released, however, followed by ventricular fibrillation (VF) that resisted to 1 mg/kg Lidocaine bolus IV administration. FHR was normal (145 bpm) at this moment. An immediate 10 J defibrillation with internal pads restored mother's sinus heart rhythm at 87 bpm. Two minutes later, severe fetal bradycardia (104 bpm) was recorded. In response, CPB FiO_2_ was increased to 100% and blood flow from 4 to 5.2 L·min^−1^. Also a bolus of 100 microgrammes (*μ*g) of phenylephrine was administrated in order to sustain a MAP superior to 70 mmHg. Maternal temperature was 36.1° at this very moment. Despite these measures, FHR increased slowly to 114 bpm which was still considered as a fetal distress sign. A low dose of nicardipine (0.5 milligrammes) was administered in order to optimize uterine blood flow by decreasing systemic vascular resistance (SVR). A minute later, fetal heart rate returned to normal (137 bpm). The patient was weaned from CPB without any difficulties and heparin was neutralized slowly with Protamine (1 mg/1 mg). Moderate anemia (Hb 8.4/dL) was managed with transfusion of one unit of packed red blood cells and the cell salvage.

Total operative time was 3 hours and 12 minutes; cross clamp time and total CPB time were 47 min and 86 min respectively.

Surgery concluded uneventfully and the patient was discharged on postoperative day 7. Histopathologic examination of the mass, confirmed the initial hypothesis of atrial myxoma (A 7 cm × 6 cm mass). Her pregnancy remained viable and she delivered a full-term healthy baby boy (Apgar score of 10) at 39 weeks GA. A two-year follow-up displayed excellent physical status of both mother and child.

## 3. Discussion

Myxoma is extremely rare during pregnancy, with very few cases having been reported in the literature. The diagnosis and management are challenging and vary depending on the volume of the mass, timing of delivery and can compromise maternal and fetal outcome [2]. Although cardiac surgery is largely practiced in our department, this is our first case of open heart surgery under CPB in a pregnant woman. The major issues in our opinion, that may have potentially adverse effects on the fetus in this case, deserve specific consideration.

The clinical presentation of this case was uncommon and the diagnosis was fortuitous thanks to the general practitioner's clinical experience. Despite the large dimensions of the tumor (7 cm × 6 cm), in accordance with the usual dimensions described in the literature, our patient was almost asymptomatic. The patient's only complaint was palpitations due to sporadic well tolerated ventricular extrasystoles, which are common during pregnancy. In more than 50% of pregnant women investigated for palpitations, ectopic beats and non-sustained arrhythmia were discovered [5, 6]. A review of hospital admissions for cardiac arrhythmia in pregnancy showed sinus arrhythmia (tachycardia/bradycardia) in 60%, premature (atrial/ventricular) contractions in 19%, supraventricular tachycardia in 14% and rare cases of atrial fibrillation (AF) in the absence of underlying heart disease (1%) [7, 8].

Whereas palpitations are rather common in pregnancy, however they are very uncommon as an isolated symptom in case of myxoma. In fact, 79.5% of patients present with one or two of the Goodwin's triad, mainly circulatory due to intra-cardiac obstruction, embolic and constitutional symptoms [2, 9] that closely correlate with patients NYHA class and the valve area [10].

Most of these symptoms (51.4%) were reported in the second trimester of gestation [2]. Given the risk of misinterpretation and consequent later decompensation in the peripartum period, the significant hemodynamic changes associated with pregnancy are important to take in consideration [11].

Diagnosis of myxomas is based on TTE, which is highly sensitive in this context [12]. Moreover, magnetic resonance imaging (MRI) is especially valuable in the diagnosis of myxomas when masses are equivocal or suboptimal on echocardiography or if the tumor is atypical in presentation and can delineate the extent of the tumor and its relationships to surrounding structures [13]. In our case, TTE images were considered sufficient to proceed to semi-urgent surgery, because of the high embolic risk for the patient.

Management strategies must take in consideration both maternal and fetal morbidity and mortality risk. Reported cases include pregnancy termination (13.7%), tumour resection during pregnancy, or delayed cardiac surgery until after delivery to guarantee fetal maturity [14]. Currently, when surgical treatment during pregnancy is indicated and can be scheduled, it is preferable between 19 and 28 weeks, to avoid the teratogenic risk during the first trimester and the risk of miscarriage after 28 weeks GA [12]. In a recent case series, cardiac surgery was mostly performed in third and second trimester in 47.2% and 38.9% respectively with a mean of 25.2 ± 9.4 GA [2].

Nevertheless, the optimal timing of surgical resection should be individualized to each patient, based on the maternal hemodynamic status and the embolic risk. Predictors of adverse maternal outcomes include NYHA class >3 and severe mitral stenosis (valve area <1.5 cm^2^, gradient >60 mmHg) [11], and are related to a mortality rate as high as 5% [10], indicating surgery during pregnancy. Those factors are also independent predictors of neonatal complication [4, 15]. In our case, the multidisciplinary team took the decision to proceed to semi-urgent surgical treatment at 19 GA because of the tumor's size and the related imminent risk of embolization or obstructive shock due to further tumor growing, despite the NYHA I status. Indeed, some authors have reported serious maternal and fetal complications during pregnancy related to embolization. Wang et al. [16] reported two pregnant patients with a cardiac myxoma complicated with cerebral infarctions requiring an urgent resection with later fetal loss. Kim et al. [17] described a case with both cerebral infarction and central retinal artery occlusion, managed by urgent surgical resection.

Cardiac surgery during pregnancy involving CPB is possible and associated with favorable maternal outcomes as reported by latest case-series. The mortality rate is similar to that of the nonpregnant women, varying from 3% to 15%, mostly on emergency [2, 4]. In contrast, CPB is associated with particularly high fetal mortality (14.3–38.5%), associated with urgent, high-risk surgery, maternal comorbidity, and early gestational age [18, 19]. To minimize fetal loss risk, surgery should be avoided in early GA if possible and CPB must be conducted according to current recommendations.

CPB can induce utero-placental hypoperfusion which may be translated into low fetal cardiac output, hypoxia and fetal loss [20]. So far, fetal circulation during CPB has not been well elucidated. Animal studies have demonstrated that utero-placental perfusion is analogous to the normal pulmonary perfusion, implying a high flow and low resistance state. Lacking autoregulation, as the vessels are widely dilated during pregnancy, the uterine blood flow, and consequently the placental blood flow, is then directly proportional to the maternal mean arterial pressure and inversely proportional to the uterine vascular resistance [21]. Maternal hypotension occurs frequently soon after the initiation of CPB, due to the systemic vascular resistance decrease, affected by hemodilution and vasoactive substances release. The resulting utero-placental hypoperfusion may be responsible for fetal hypoxia and subsequent fetal bradycardia. Being an important indicator of fetal distress during CPB, fetal bradycardia is known to develop frequently upon its initiation and the rythm normalizes after the end of the procedure [21]. Other reported causes are: uterine contractions also precipitated by utero-placental hypoperfusion, maternal hypothermia, hypoglycaemia, administration of drugs to the parturient that cross the placenta (opioids, propranolol), rapidity of initiating CPB, type of myocardial protection and the ischemic time [22].

In order to provide adequate utero-placental perfusion and to guarantee fetal protection [23, 24], current recommendations suggest: high perfusion pressure (>70 mmHg), high flow (2.5 L/min/m^2^), and pulsatile, normothermic CPB [19, 22]. Maternal hematocrit >28% is also recommended for optimizing oxygen transfer [23] and a-Stat pH management, in order to protect the fetus against hypocapnia and uteroplacental vasoconstriction [4].

Our CPB management was conducted according to these recommendations, using continuous fetal heart rate monitoring (FHR). Neither maternal hypotension, nor fetal bradycardia were observed in the EFM patterns during the progressive transition from systemic to driven perfusion.

Monitoring of FHR during CPB is important, since it has been reported to reduce fetal mortality to 9.5% by enabling early recognition of fetal heart dysfunction and prompt delivery of adequate treatment [25]. A qualified practitioner to interpret the tracing is mandatory, as well as a preoperatively established strategy with regard to how an abnormal tracing should be anticipated by the team [26]. Considered currently the standard of care, it should be focused to maintain FHR between 110 and 160 bpm and guide the perfusionist to adjust the perfusion flow rate, the mean arterial pressure, and the maternal temperature.

FHR monitoring can be easily applied during cardiac surgery using an external cardiotocograph (EFM), as reported by several case series [25]. However, when applied before 24 GA, technical difficulties related to the small size of fetus and its unpredictable position during the operation may compromise the continuity and accuracy of EFM pattern.

In our case, despite the small fetal size, the attending midwife obtained continuous FHR monitoring by moving during surgery the cardiotocograph probe over the abdomen and confirmed a normal fetal heart rate. Intriguingly, bradycardia was observed immediately following the internal electric shock delivered to treat ventricular fibrillation after aorta unclamping, under stable CPB maternal hemodynamic conditions.

External electrical cardioversion as a treatment for serious maternal dysrhythmias during pregnancy is generally safe for the fetus. Many reports advocated that standard energy current applied by carefully placed pads to avoid it's transmission to the uterus, does not affect the fetal heart [27]. Nevertheless, in 2002, Barnes et al. reported a case of important fetal bradycardia following external defibrillation for supraventricular tachycardia at 37 GA [28]. An emergency caesarean delivery was performed and the uterus was found tightly contracting. The author suggested that the current could have reached the enlarged uterus and provoked uterine contractions, thus leading to fetal distress. Concerning defibrillation in the parturient during CPB, research data and clinical experience in this area are limited.

We are aware of only two available clinical cases using a single 10 joules shock to restore sinus maternal rhythm [22, 29]. In both reports, fetal bradycardia occurring shortly after the beginning of CPB, sustained after defibrillation, however, without being related to it by the authors. Interestingly, in their report Mahli et al., mentioned also the occurrence of uterine contractions intraoperatively and the administration of nitroglycerin as tocolytic treatment [21].

Although the causes are still unclear, uterine contractions occur frequently during CPB. Importantly, they can reduce uterine blood flow by increasing vascular resistance, resulting in feto-placental hypoperfusion and fetal distress [30]. Thereby, intraoperative uterine monitoring is recommended [3, 4, 11, 22]. In our case, unfortunately, intraoperative uterine activity was not monitored because of the early GA. However, when fetal bradycardia occurred we hypothesized that it could have been caused by defibrillation related uterine contractions. Considering that fetal EFM patterns were optimal throughout CPB until that moment, we suppose that it was unlikely to be related to mother's hemodynamic state or to lidocaine's IV administration. Furthermore, a correlation between lidocaine and fetal bradycardia had been suggested only by a single animal study [31].

Immediately upon fetal bradycardia, our strategy consisted primarily in increasing pump flow and sustaining the MAP ≥ 70 mmHg, as recommended [32], by a short acting vasoconstrictor. Since the FHR did not recover after these means, we then administered a very low dose of nicardipine as a tocolytic treatment, which was followed by FHR recovery.

Tocolytic therapy, mainly with *β*2-agonists, has been successfully used to stabilise uterus during CPB [21, 33], however, it still remains under debate. Also, nitroglycerine, a vasodilator, has been commonly used to lower placental vascular resistance and improve placental blood flow; however, its use in some recent case reports was not beneficial for the fetus [22, 29]. Our choice of using a calcium channel blocker, was based on its properties of being an arteriolar vasodilator acting favourably on uteroplacental circulation together with direct uterine contractility inhibitor. Although they have been extensively studied in pregnancy, mainly in the management of preeclampsia, studies have yielded conflicting results concerning their effects on uteroplacental circulation. Whereas uteroplacental flow decreased in animals, human studies have shown either no change or decrease in vascular resistance [34].

Consistently with the good fetal outcome following the chosen intraoperative strategy, we believe that the direct defibrillation was probably responsible for the main adverse effect on the fetus during CPB. Management of the flow and pressure together with the administration of nicardipine as tocolytic treatment, have been the adequate treatment. And even though nicardipine was administered in low dose, it seemed to be beneficial in restoring FHR in our case.

## 4. Conclusion

Myxoma diagnosis and management during pregnancy is a particularly difficult challenge and should be ensured by a multidisciplinary and collaborative approach. CPB when indicated can be performed with relative safety when based on a well-established surgery. Both routine cardiac follow-up and prenatal counselling are important aspects to preventing urgent delivery. We are convinced that reporting new cases is an important contribution to the further improvement of maternal and fetal care.

## Figures and Tables

**Figure 2:**